# Assessment of sealing ability, antibacterial effect and marginal adaptation of bioactive materials in pulpotomized primary molars: an in-vitro study

**DOI:** 10.1186/s12903-025-07563-1

**Published:** 2026-01-17

**Authors:** Reham Ahmed El-Nemr, Wedad M. Nageeb, Noha El-Sayed Fathi Abdou

**Affiliations:** 1 Pediatric Dentistry, Preventive Dentistry and Dental Public Health Department, Faculty of Dentistry, Suez Canal University, Ismailia, Egypt; 2 Medical Microbiology and Immunology Department, Faculty of Medicine, Suez Canal University, Ismailia, Egypt; 3 Pediatric Dentistry, Preventive Dentistry and Dental Public Health Department, Faculty of Dentistry, Aswan University, Aswan City, Egypt

**Keywords:** Biodentine, Conofocal laser microscopy, Enterococcus faecalis, Environmental scanning electron microscope, MTA, NeoPUTTY

## Abstract

**Background:**

The success of pulpotomy depends on the restorative material’s ability to provide an effective seal, preventing bacterial penetration and growth within the root canal. This study was performed to compare three different types of calcium silicate-based cement in pulpotomized extracted primary molars considering antibacterial effect, sealing ability and marginal adaptation.

**Materials and methods:**

Recently extracted second primary molars were randomly categorized into three experimental groups (*n* = 10 each). Pulpotomy cavity preparations were made and pulp dressing materials were placed as follows: MTA (group I), Biodentine (group II) and NeoPUTTY (group III). All teeth were contaminated with *Enterococcus faecalis* and incubated for 21 days. One half of each molar was stained with the LIVE/DEAD BacLight Bacterial Viability Kit and evaluated using Confocal Laser Scanning Microscopy (CLSM) for observing live and dead bacteria based on fluorescence. The other half of each molar was used to measure the gap between pulp dressing material and the dentine walls using high-resolution Environmental Scanning Electron Microscopy (EESEM).

**Results:**

The tested bioactive materials significantly influenced the total bacterial count, with MTA showing the highest bacterial leakage when compared to both Biodentine and NeoPUTTY *(p = 0.008, p = 0.012)*, respectively. The mean live bacterial load in the MTA group was significantly higher than in the Biodentine and NeoPUTTY groups *(p < 0.0001)*. Microgap width of interfaces between MTA and dentine (9.88 ± 4.12) was significantly larger in comparison to NeoPUTTY and (7.44 ± 1.46) *(p = 0.006)*, however the difference was non-significant compared to Biodentine (8.84 ± 3.99) *(p = 0.356)*.

**Conclusions:**

All the tested bioactive materials effectively inhibit the growth of *bacteria*. Biodentine and NeoPUTTY are promising and show favorable results for use as coronal pulpotomy dressing material owing to superior antibacterial activity, sealing, and marginal adaptation relative to MTA.

## Introduction

The premature loss of primary teeth may result in adverse consequences, including compromised masticatory function, loss of space, malocclusion, unusual tongue habits, speech impediments, psychological issues and consequently affecting the growth and development of the child. Therefore retention of primary teeth until natural exfoliation is crucial [1, 2].

Pulpotomy is the preferred treatment for severely carious primary teeth or following iatrogenic pulp exposure in asymptomatic primary teeth or those with reversible pulpitis, which would otherwise necessitate extraction. The procedure aims to preserve the vitality of the radicular pulp after surgical removal of the infected coronal pulp by placing a pulpotomy base material (PBM) followed by permanent restoration [3, 4]. A major cause of failure is coronal microleakage, which allows bacterial penetration. For successful outcomes, PBMs should have good bonding with the pulp dentin complex and the upper final restoration, ensuring an effective seal to prevent postoperative sensitivity, secondary caries, and bacterial proliferation [5, 6].

Tricalcium silicate-based cement has been used in pulpotomy due to their ability to integrate with host tissues and promote favorable biological responses [7]. Among these, mineral trioxide aggregate (MTA) has demonstrated excellent biocompatibility, sealing capability, and antibacterial properties. It can activate fibroblasts in primary teeth, stimulating cytokines secretion that facilitate hard tissue formation, hence possessing the potential to enhance tissue regeneration during pulp therapy [8, 9]. Reported success rates of MTA pulpotomy range from 88.2% to 100% [10–12]. Biodentine has been marketed as a “bioactive dentin substitute” with superior biological and physical properties compared to MTA [13]. NeoPUTTY, a premixed bioceramic putty, was introduced to overcome some drawbacks of MTA such as prolonged setting time, potential discoloration, and handling difficulties. It contains tantalum oxide, dicalcium silicate, and tricalcium silicate, which promote hydroxyapatite formation and healing, and sets upon contact with moisture [14].

Several methods have been used to assess leakage, bacterial leakage test is considered more clinically relevant than dye or fluid-based techniques [15, 16]. Furthermore, various microscopic methods, including stereomicroscopy, scanning electron microscopy (SEM), and transmission electron microscopy (TEM), have been used to assess bacterial colonization on dentin, however, these methods primarily serve descriptive functions, being indirect and non-quantitative, and are incapable of assessing bacterial viability. Thus, confocal laser scanning microscopy (CLSM) has been employed to provide a direct, non-destructive, three-dimensional evaluation of bacterial viability within dentinal tubules by dead/live staining techniques, offering more clinically relevant results than conventional microleakage models [17–19]. In addition, in the present study, environmental scanning electron microscopy (ESEM) was employed to detect interfacial gaps between dentin and pulp capping materials.

To date, no study has evaluated the bacterial sealing ability and viability of *Enterococcus faecalis* in pulpotomized primary molars treated with NeoPUTTY using advanced imaging techniques including CLSM and ESEM. Unlike previous research on MTA and Biodentine, which relied mainly on conventional microbiological methods, this study provides the first comprehensive histological assessment of NeoPUTTY, Biodentine and MTA under controlled bacterial challenge within the central, mesial, distal, and apical areas of the pulp chamber following pulpotomy, filling a key gap in the literature. This study aimed to compare the sealing ability and antibacterial efficacy of NeoPUTTY, Biodentine, and MTA using a bacterial leakage model with *E. faecalis*. CLSM was employed to assess bacterial colonization and viability across different pulp chamber regions. This dual-method approach offers new insights into the biological and structural sealing behavior of these materials under standardized conditions. Our null hypothesis stated that there would be no significant differences in total bacterial loads, viability, or interfacial gaps between the three materials.

## Materials and methods

Ethical clearance was obtained from the Research Ethics Committee (REC) of the Faculty of Dentistry at Suez Canal University, with permission number (881/2024), in accordance with the principles of the Helsinki Declaration. Informed consent was obtained from parents/legal guardians for the use of extracted teeth. Forty freshly extracted second primary molars (for orthodontic purposes) were collected. Only mandibular second primary molars with sufficient tooth structure, an intact pulpal floor, and at least three intact axial walls were included. Teeth must not have undergone any previous pulp therapy and were required to have at least one-third of the root structure remaining. Molars with extensive caries, cracks or restorations were excluded [20].

### Sample size calculation

Using G*Power 3.1.9.6, a minimum of 30 samples was required to detect an effect size of 0.27 with α = 0.05 and β = 0.2 (power = 80%, partial eta²=0.07). To ensure validity, 40 teeth were included [21].

### Sample preparation

Teeth were cleaned, polished with pumice, autoclaved at 121 °C for 20 min to ensure internal sterility prior to cavity preparation, and stored in sterile saline until the commencement of the experiment [22]. 

Standardized pulpotomy cavities were prepared using #330 and #6 carbide burs *(Komet*,* Lemgo*,* Germany)* under water spray. The teeth were then air dried, and a digital caliper was employed to verify a standardized access cavity diameter of 3 mm x 3 mm [16]. Pulp remnants were removed with K-files up to size 30 (Mani, Japan). During preparation, 5 ml of 2.5% sodium hypochlorite (NaOCl; JK-Dental, Egypt) was used as an irrigant for 1 min, followed by 5 ml of 17% EDTA (Promega, USA) for 1 min to eliminate residual chemical debris [23]. Teeth were disinfected in 2.5% NaOCl for 24 h to eliminate potential surface contamination introduced during handling or preparation, then rinsed and dried with size 30 paper points (Meta Biomed, South Korea) and a small cotton pellet (Roeko, Coltene, Germany) [16].

### Sample allocation

Thirty primary mandibular second molars indicated for pulpotomy were sequentially numbered from 1 to 30 and randomly allocated into three groups (*n* = 10 each) using an online randomization tool (www.randomizer.org), according to the type of base material applied; Group I (*n* = 10): MTA (Dentsply, USA), Group II (*n* = 10): Biodentine (Septodont, France) and Group III (*n* = 10): NeoPUTTY (NuSmile, USA). Additionally, ten teeth served as controls: negative (intact, *n* = 5) and positive (pulpotomized but unfilled, *n* = 5). Materials were prepared per manufacturer’s instructions and placed to half of the coronal height. All teeth were preserved in a sterile incubator (Memmert GmbH + Co. Schwabach) at 37 °C and 100% humidity for 24 h to ensure complete material setting, subsequently stored in distilled water at 37 °C for an additional 24 h to simulate clinical conditions and allow continued hydration, maturation, and development of desirable microstructure/sealing ability [16, 24].

### Experimental bacterial leakage model and *Enterococcus faecalis* bacterial contamination

Two layers of nail varnish were applied to the root surfaces of all samples to inhibit bacterial leakage through lateral canals or other irregularities in the cementum. Subsequently, all teeth were affixed utilizing a sterile microleakage model adopted from previous studies [15, 16, 25]. Each tooth was mounted in a 1.5 ml sterilized plastic microtube and then fitted into a sterilized glass vial and sealed with a cyanoacrylate adhesive *(3 M*,* US)*, followed immediately by bacterial contamination [15, 25]. Enterococcus faecalis (ATCC^®^ 19433) suspension was freshly prepared and placed in the upper chamber of the microtube on Day 1 and replenished every 48 h. to ensure viability of bacteria. Models were incubated at 37 °C in 100% humidity for 21 days [15, 23, 25].

### Preparation of samples for evaluation

After 21 days, teeth were embedded in acrylic resin *(Triad VLC resin; Dentsply*,* York*,* PA)* and sectioned mesio-distally through the pulp chamber and furcation using a diamond saw *(Isomet*,* Buehler*,* USA)* rotating at 500 rpm under water cooling [15].

### Confocal laser scanning microscopy (CLSM)

Half of the specimens from each group were stained using the LIVE/DEAD BacLight Bacterial Viability Kit L-7012 *(Molecular Probes*,* Eugene*,* OR*,* USA)*, containing separate vials of the two component dyes; SYTO 9 (green-fluorescent nucleic acid stain) and Propidium Iodide (red-fluorescent nucleic acid stain) in a 1:1 mixture. The excitation/emission maxima were 480–500 nm for SYTO 9 and 490–635 nm for propidium iodide. These dyes selectively stain live (intact membrane) and dead (damaged membrane) bacteria, respectively. Then confocal microscopy evaluation was performed immediately after the staining procedure.

Immediately after staining, the confocal laser scanning microscope *(Leica Microsystems DMi 8*,* CMS GmbH)* was used to observe the stained bacteria. Each specimen was subjected to deep scans performed in a format of 1024 × 1024 pixels to evaluate the central, mesial, distal and apical areas based on anatomical landmarks. The central region was located at the middle of the pulp chamber floor, mesial and distal regions corresponded to the respective pulp horns, and the apical region was defined adjacent to the root canal orifices. Fluorescence intensity was measured as an average of five random fields within the defined boundaries of each region for each sample to assess the degree and amount of bacterial penetration. The vitality of the bacteria was determined according to percentage of live and dead bacteria, and the type of sealing material were assessed [15, 16, 25]. Image processing and analysis was performed using Fiji (ImageJ 2.x) [26]. All measurements were carried out by a single well trained examiner who was blinded to the type of material used to eliminate bias.

### Environmental scanning electron microscope (ESEM)

The remaining half of specimens were immersed in varying concentrations of aqueous ethanol—70%, 80%, 90%, 95%, and 100%—for 5 min each to eradicate any residual bacteria, facilitating the examination of the gap and spaces between materials and tooth structure using an environmental scanning electron microscope [16, 27].

**Fig. 1:**
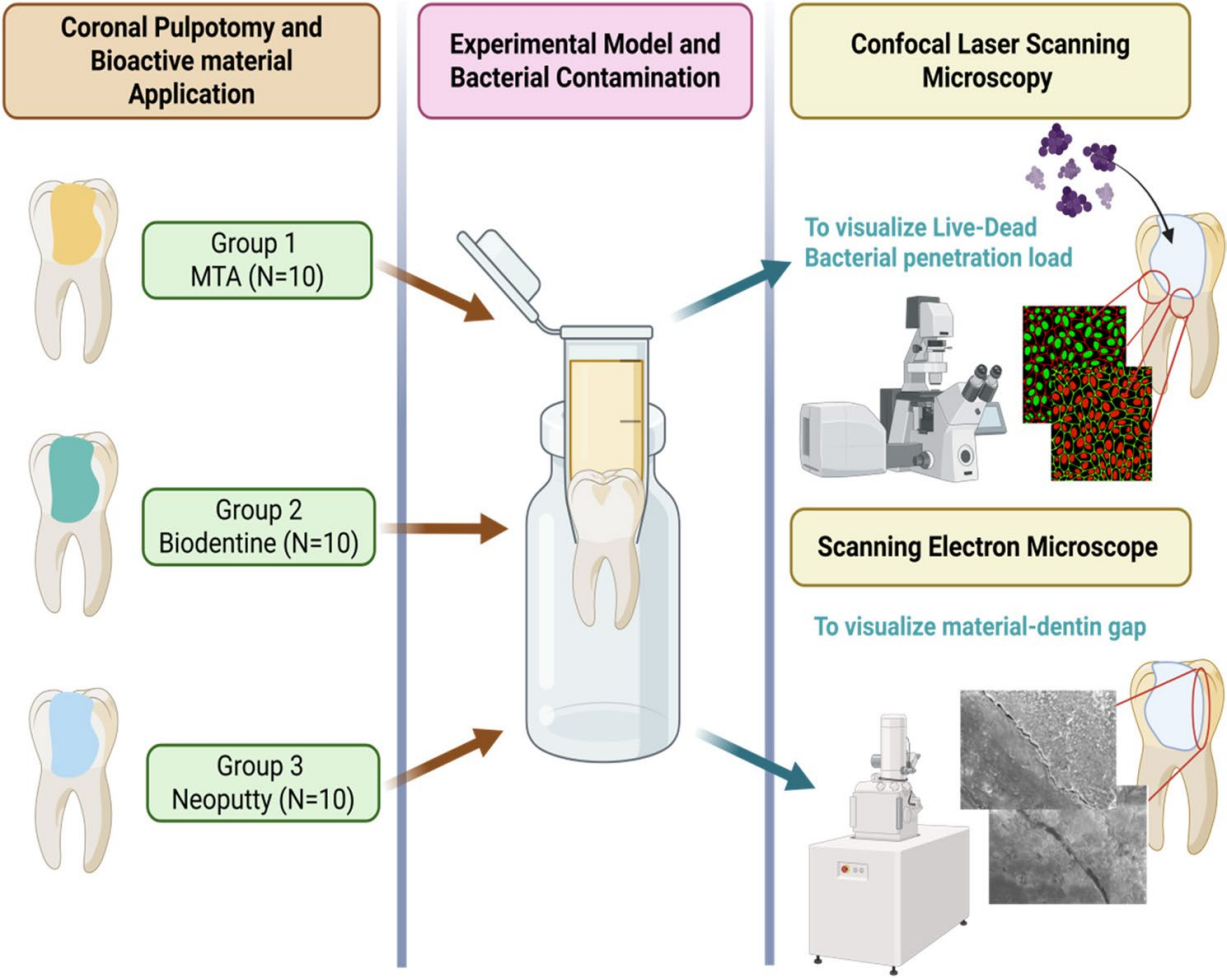
Workflow showing experimental steps performed

The marginal adaptation between the pulp capping materials and cavity dentin walls was evaluated using a high-resolution ESEM *(Quanta FEG 250*,* FEI*,* USA)* operated at 20 kV. Images were obtained at 1000× and 2000× magnifications, and multiple points (mesial, pulpal floor and distal) along the material–dentine interface were analyzed using ESEM to assess interfacial gaps and marginal adaptation [28]. All measurements were performed by a single calibrated examiner who was blinded to the type of material used to eliminate bias. Summary of the experimental workflow performed is provided in Fig. 1.

### Statistical analysis

Data entry was performed using Microsoft Excel sheets for analysis. Mean and Standard Deviation were used to describe normally distributed data. Shapiro-Wilk test was used to test for normality of data distribution. One-way ANOVA with Tukey Post-hoc comparison was used to compare tested groups to assess the difference in bacterial penetration/leak through pulp capping material and the percent of dead and live bacteria. The difference in gap area measurements between different tested groups was also assessed using One-way ANOVA. A two-way between-groups ANOVA was used to evaluate the fluorescence intensity at the apical/mesial/distal/central areas and to compare between the 3 Bio-active materials. Statistical analysis was performed using SPSS Statistical package for analysis (SPSS^®^ sofware, IBM).

## Results

### Confocal laser scanning microscopy (CLSM) results

Emission wavelengths of 505–550 nm (green, Syto9) and 650–750 nm (red, PI) were collected to visualize Syto 9 and PI, respectEmission wavelengths of 505–550 nm (green, Syto9) and 650–750 nm (red, PI) were collected to visualize Syto 9 and PI, respectively, by two detection channe.

To assess the difference in bacterial penetration/leak through pulp dressing material, total Bacterial Load inside tooth material, as shown by fluorescent intensity, was compared between different groups of materials used. Shapiro-Wilk test showed that the data were normally distributed. One-way ANOVA showed a statistically significant difference between the three groups of material (*p* = 0.005). The effect size, calculated using eta squared, was 0.686. Tukey Post-hoc testing showed that MTA demonstrated a statistically significant difference in total bacterial count with the highest Bacterial leakage for MTA when compared to both Biodentine and NeoPUTTY (*p* = 0.008, *p* = 0.012) respectively. No significant difference in bacterial leakage was found between Biodentine and NeoPUTTY (*p* = 0.972). These results are shown in Table 1.

**Table 1:** Total bacterial Leakage/penetration of the three tested bioactive materials as assessed using fluorescent intensity measured by confocal microscopy

	Mean	Std. Deviation	Std. Error	95% Confidence Interval for Mean		Minimum	Maximum
				Lower Bound	Upper Bound		
NeoPUTTY	71608167.3	18642471.4	9321235.7	41943835.3	101272499.3	5.26E + 7	9.59E + 7
MTA	154226888.5	45312672.4	22656336.2	82124315.1	226,329,462	1.10E + 8	2.06E + 8
Biodentine	66531494.6	23527674.3	11763837.2	29093714.5	103969274.6	4.51E + 7	9.97E + 7
Total	97455516.8	50679448.5	14629896.6	65255331.4	129655702.2	4.51E + 7	2.06E + 8

**Fig. 2:**
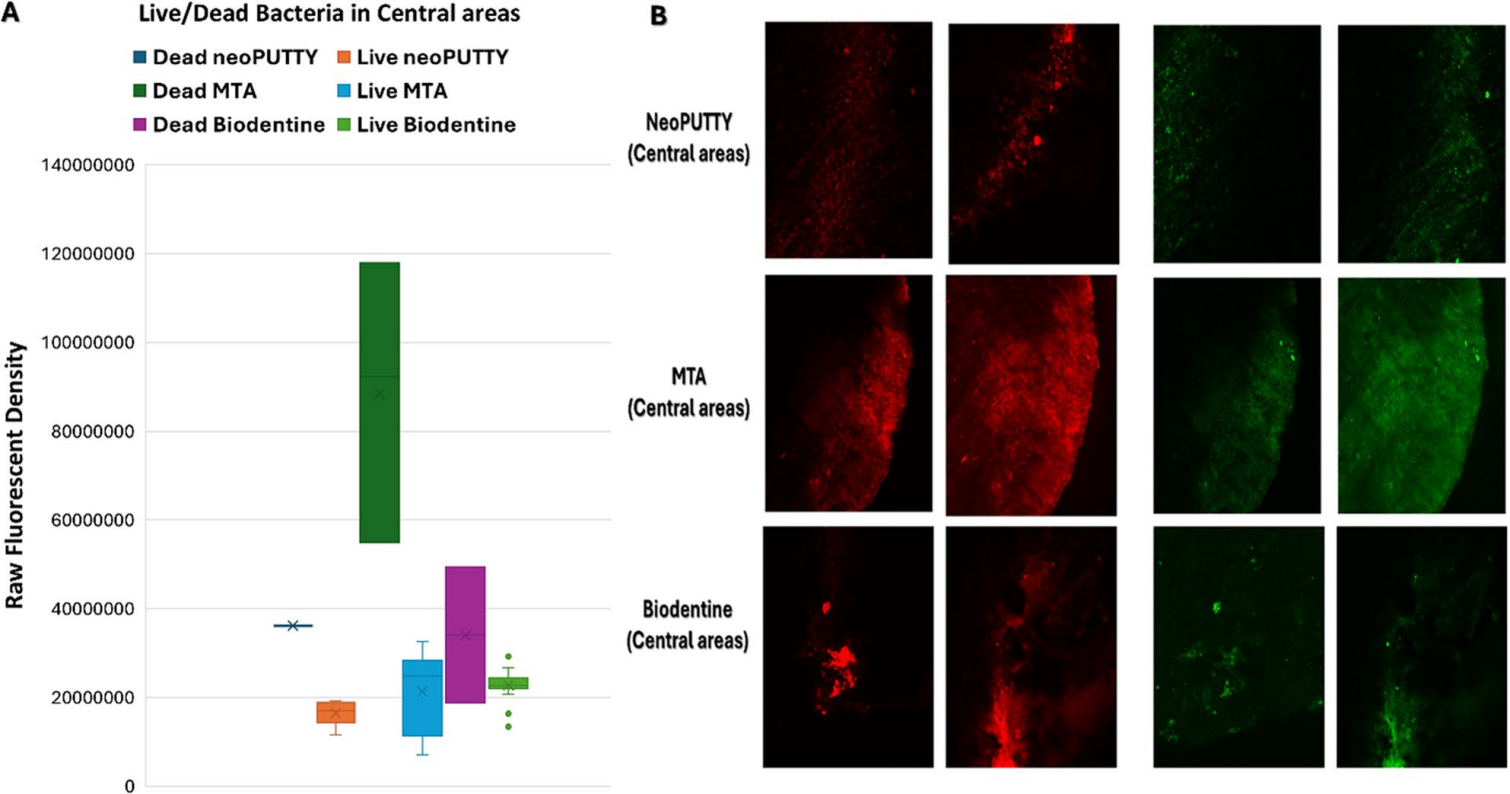
Load of Live and dead bacteria for the three studied bioactive material as represented by Fluorescent Intensity in central areas

**Fig. 3:**
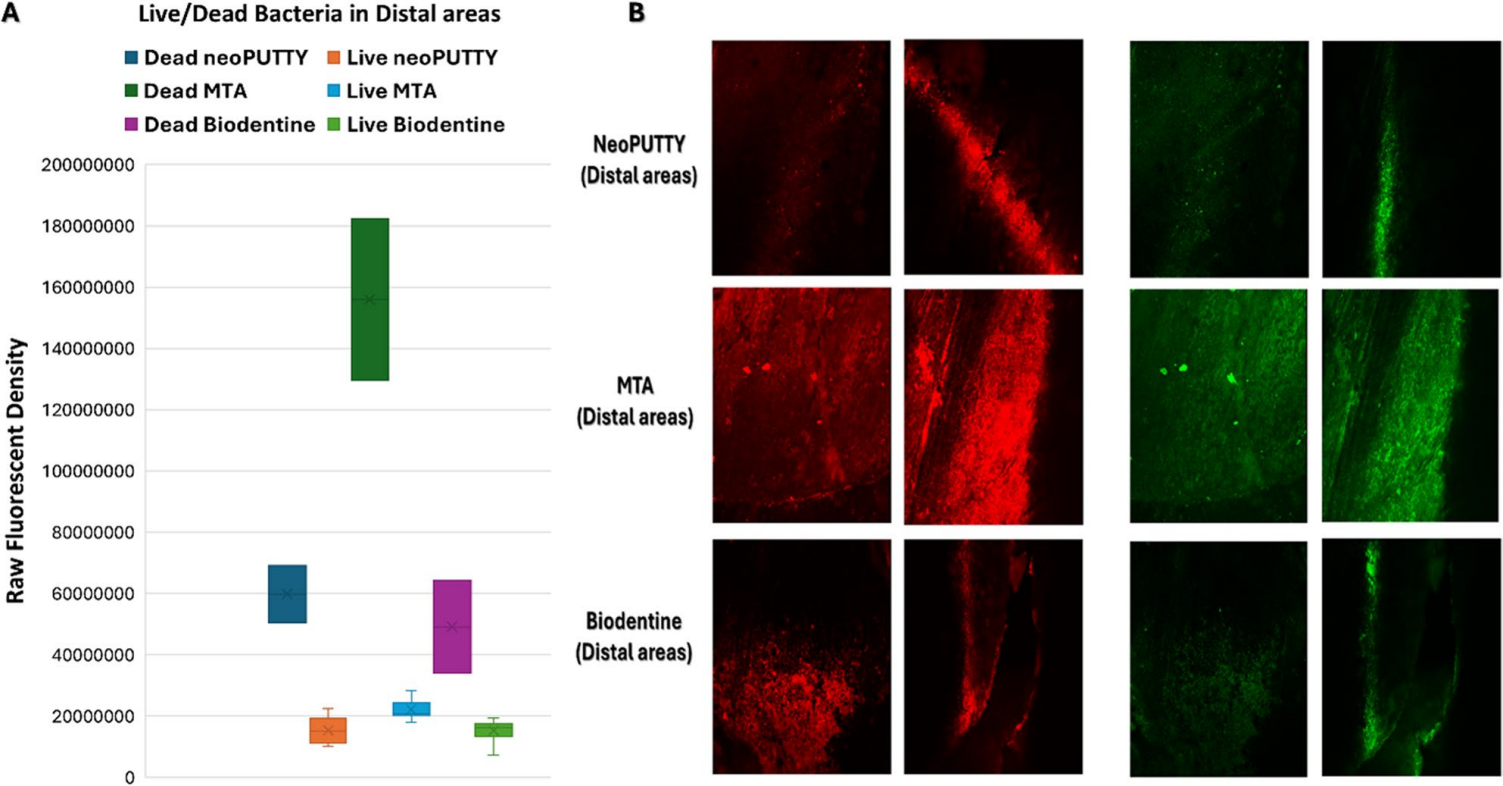
Load of Live and dead bacteria for the three studied bioactive material as represented by Fluorescent Intensity in Distal areas

**Fig. 4:**
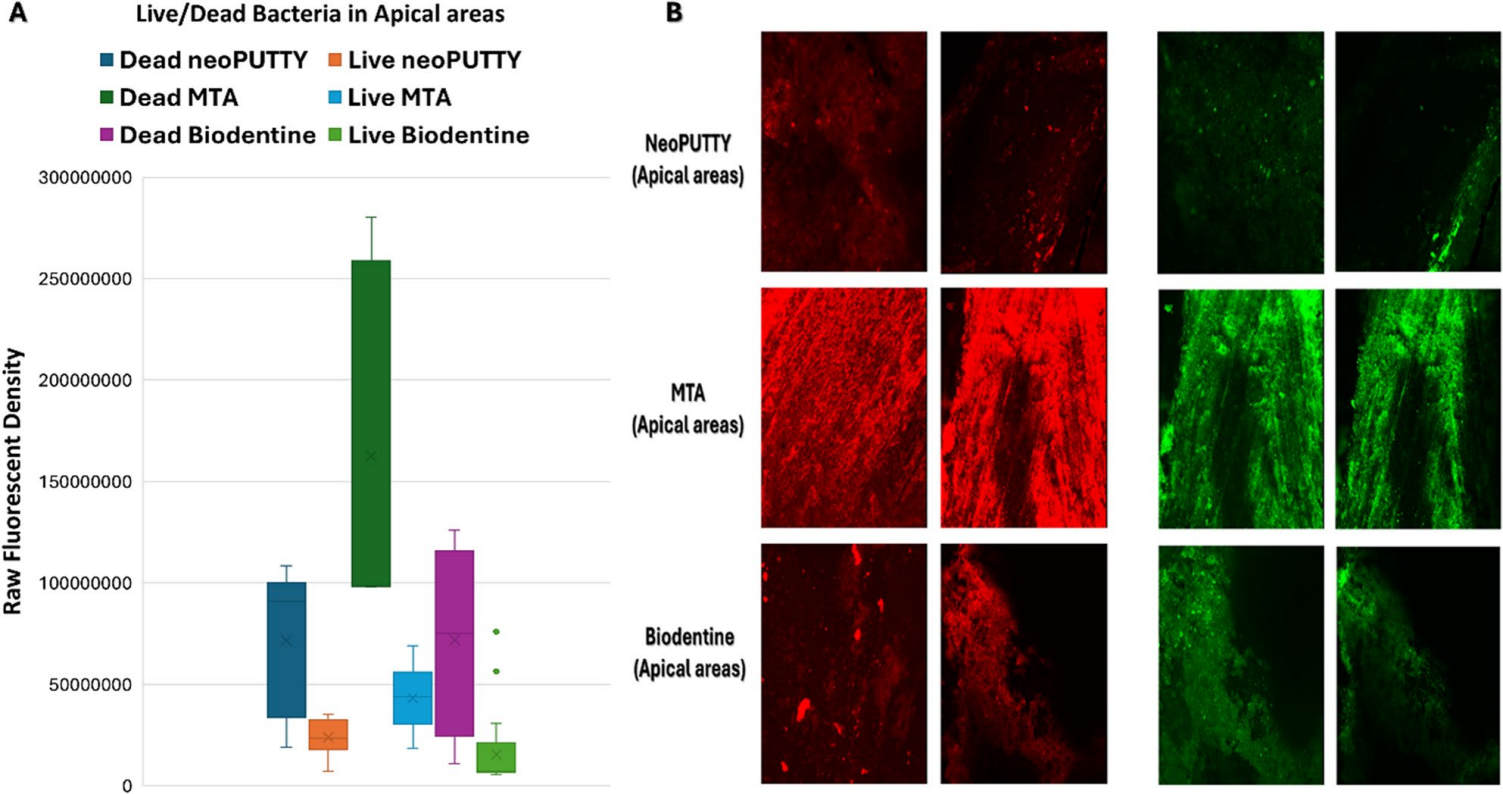
Load of Live and dead bacteria for the three studied bioactive material as represented by Fluorescent Intensity in Apical areas

**Fig. 5:**
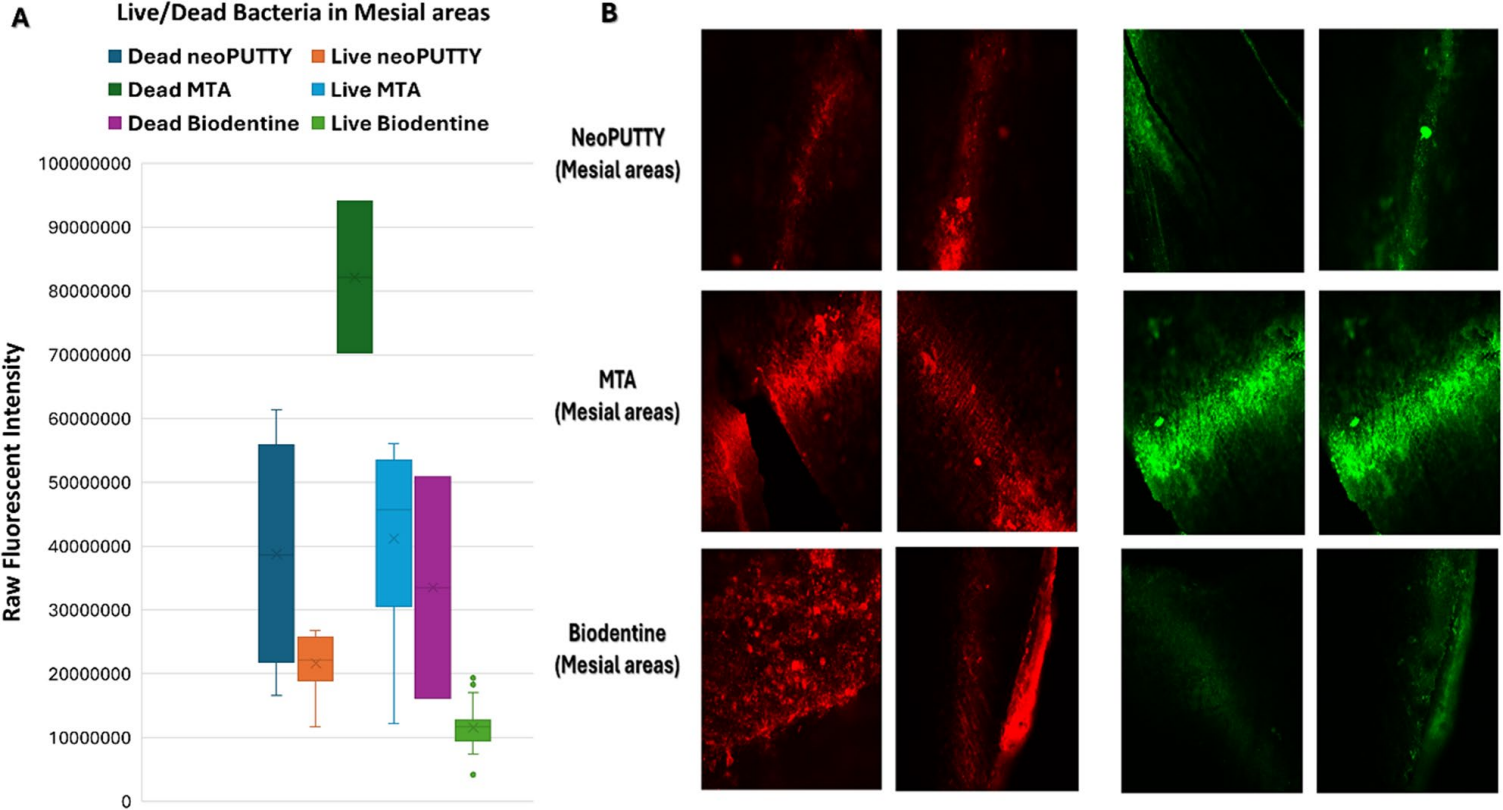
Load of Live and dead bacteria for the three studied bioactive material as represented by Fluorescent Intensity in Mesial areas

**Fig. 6:**
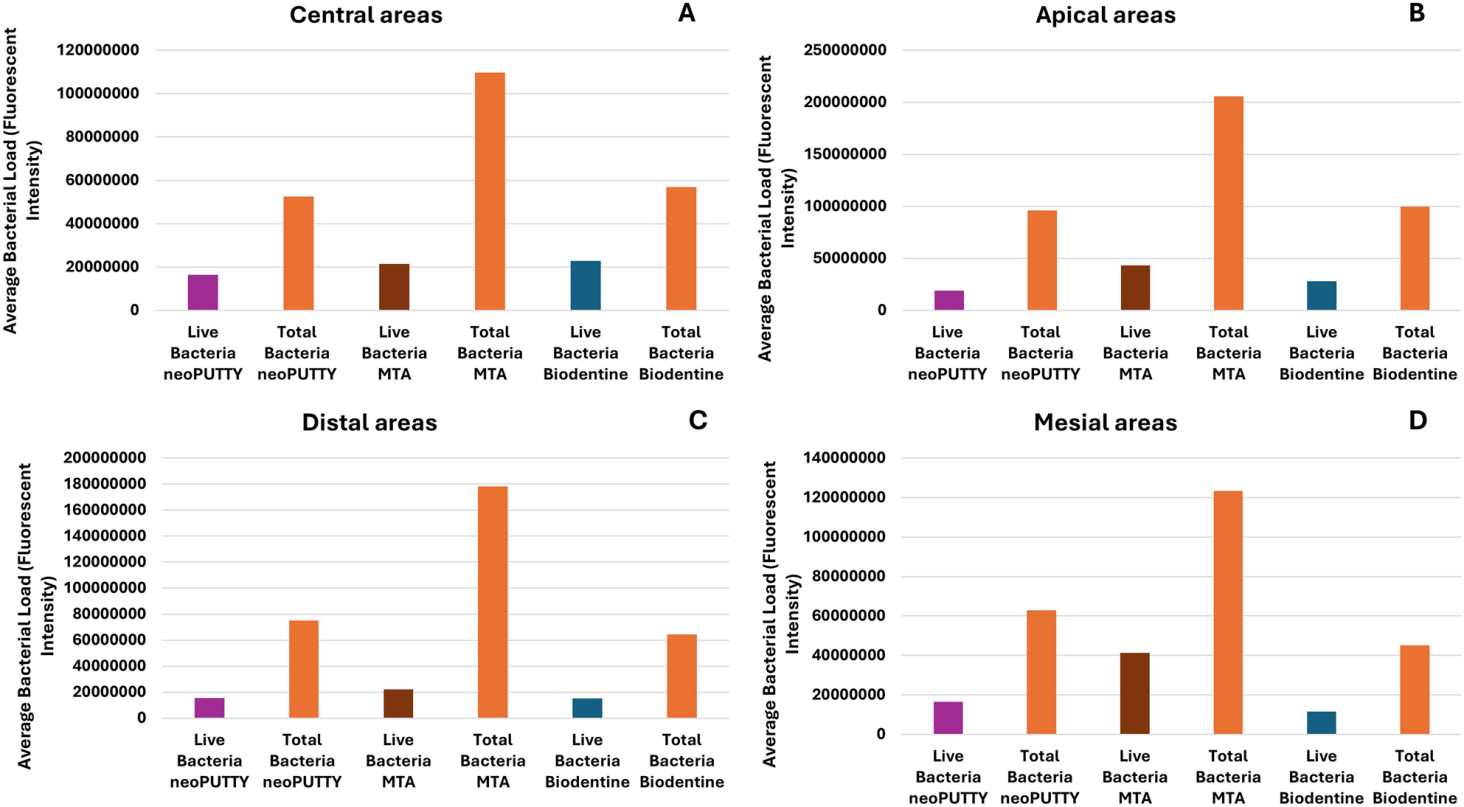
Antibacterial effect of different tested pulp capping material assessed using Confocal Microscopy at different visualized sections of examined teeth

**Fig. 7:**
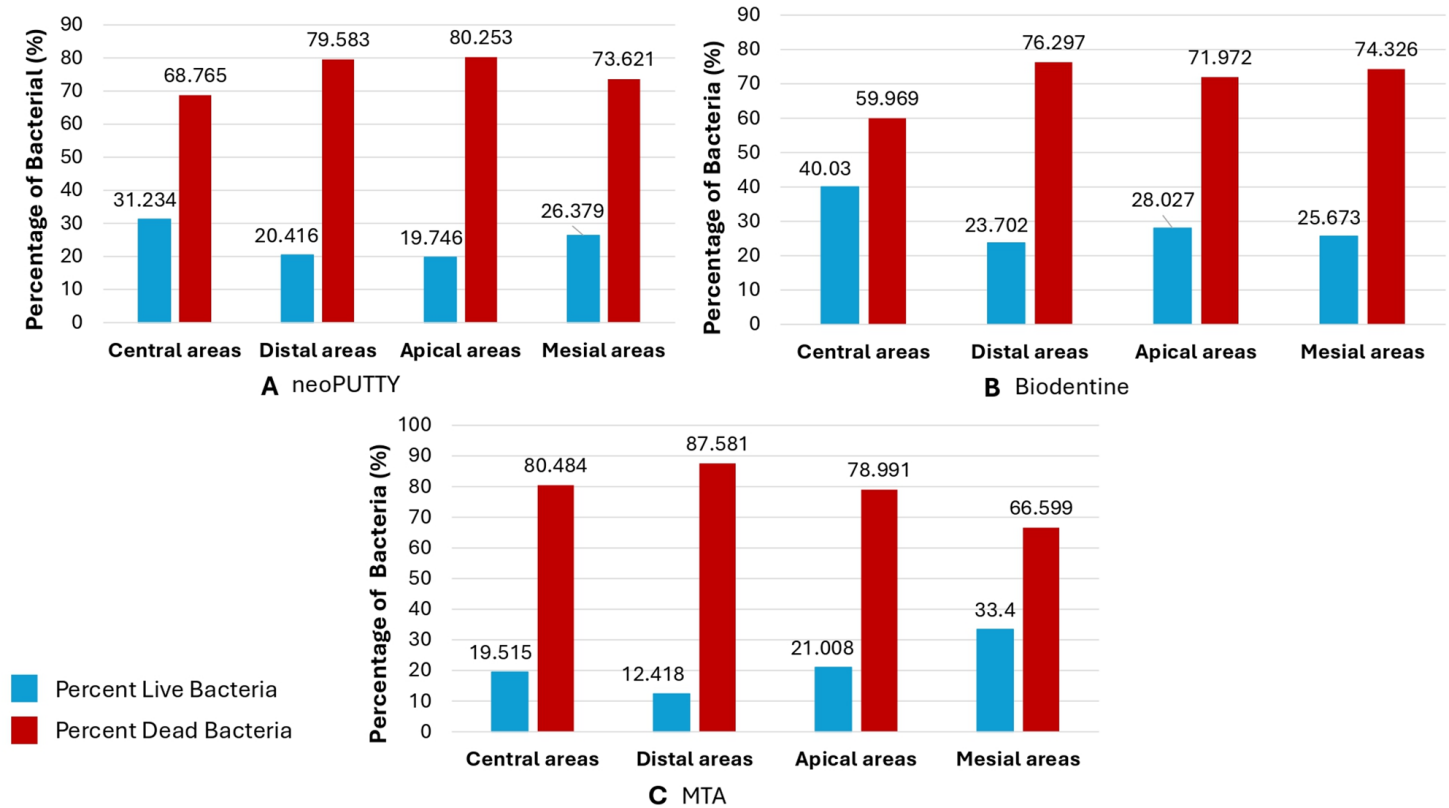
Percentage of dead bacteria compared to live bacteria among the three tested bioactive materials

The live bacterial loads in teeth treated with different pulp-capping materials were compared across the apical, mesial, distal, and central regions, as illustrated in Figs. 2, 3, 4, 5 and 6, while the percentage of dead bacteria compared to live bacteria among the tested materials is shown in Fig. 7.

A significant overall interaction effect was detected in between tested samples (*p* < 0.0001). There was a statistically significant main effect for type of pulp dressing material (*p* < 0.0001) with moderate effect size (partial eta squared = 0.369). Post-hoc comparisons using Tukey HSD test indicated that the mean live bacterial load in MTA group was significantly higher than both Biodentine and NeoPUTTY groups (*p* < 0.0001 for both). The difference between Biodentine and NeoPUTTY did not show statistical significance (*p* = 0.97) as illustrated in Table 2; Fig. 7.

**Table 2:** Mean percentage of bacterial killing by different pulp capping materials, expressed as the ratio of dead bacteria to total bacteria

	Mean	Std. Deviation	Std. Error	95% Confidence Interval for Mean		Min	Max
				Lower Bound	Upper Bound		
NeoPUTTY	75.5750	5.42609	2.71305	66.9409	84.2091	68.80	80.30
MTA	78.4000	8.72238	4.36119	64.5207	92.2793	66.60	87.60
Biodentine	70.6500	7.31414	3.65707	59.0116	82.2884	60.00	76.30
Total	74.8750	7.38624	2.13222	70.1820	79.5680	60.00	87.60

Considering the evaluated areas, results showed significant differences (*p* < 0.0001) with small effect size (partial eta squared = 0.087). Live bacterial load was significantly lower in central part compared to both apical areas (*p* = 0.05) and mesial areas (*p* < 0.0001). The mesial areas, particularly with MTA, exhibited a significantly higher live bacterial load compared to the other three regions (central, apical, and distal) (*p* < 0.0001). Conversely, the distal areas demonstrated a significantly lower live bacterial load than the apical regions (*p* < 0.0001), as illustrated in Figs. 2, 3, 4, 5 and 6).

### Scanning electron microscope (ESEM) results

**Fig. 8:**
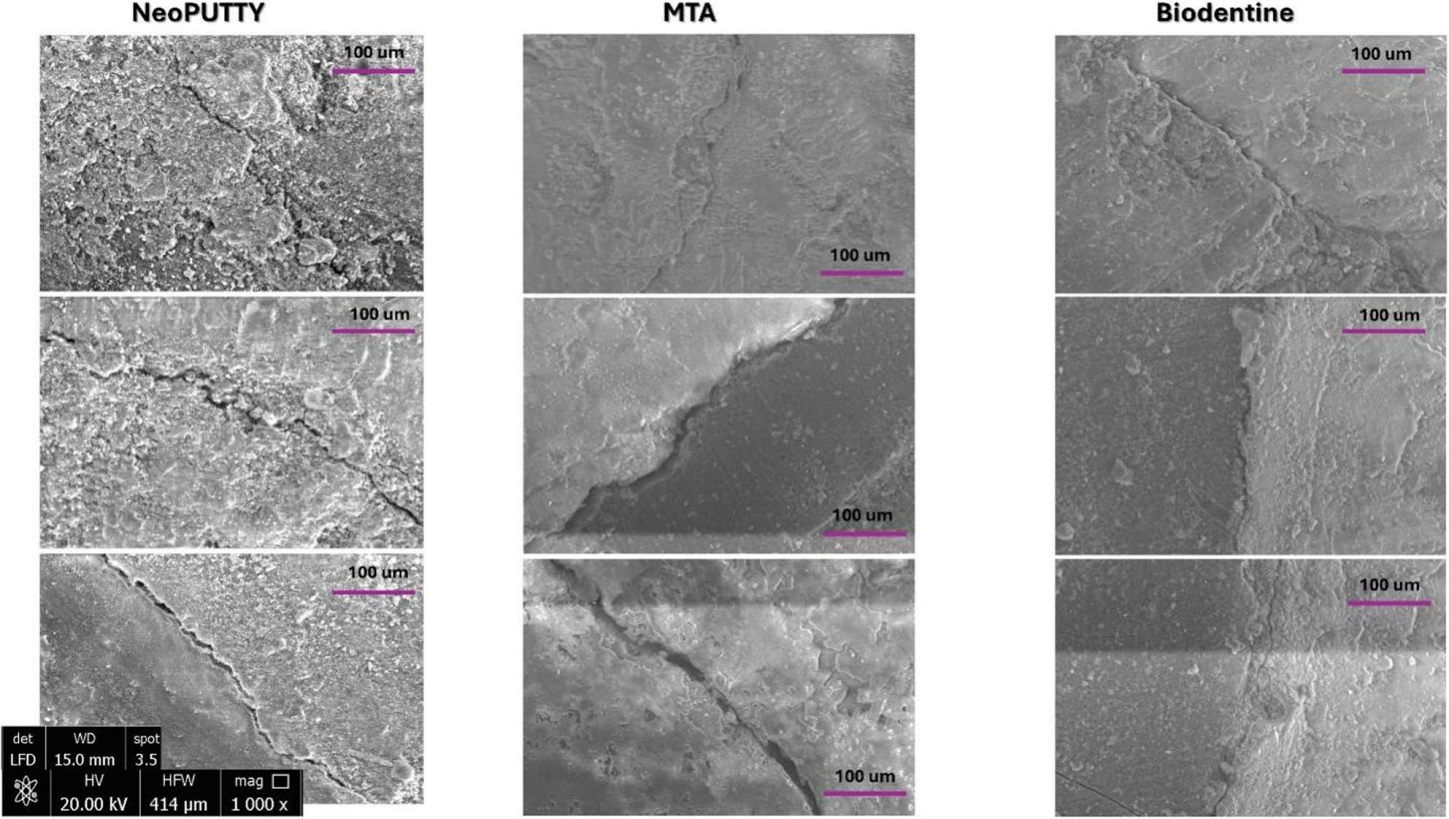
Representative SEM scanning micrographs displaying cavities filled with different pulp capping materials and representing the gap interphase between tested material and dentin

**Fig. 9:**
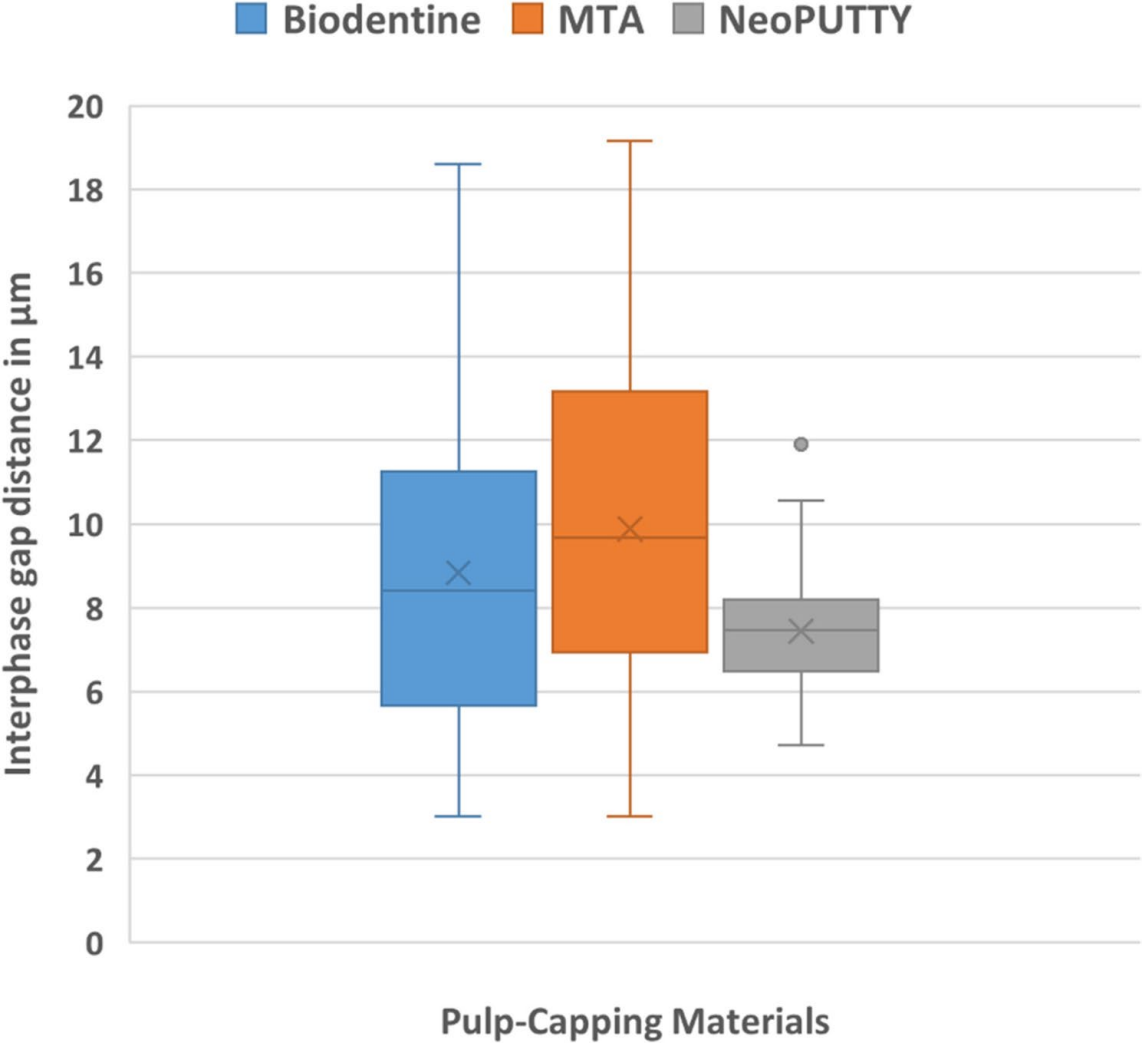
Comparison of interphase gap distance between pulp-filling material and dentin among three tested materials

The mean gap widths and standard deviations (µm) between dentin and the pulp dressing materials for each experimental group are presented in Table 3; Fig. 8, and Fig. 9. One-way ANOVA revealed a statistically significant difference in the mean microgap width between groups (*F* = 5.046, *p* = 0.008). In particular, the MTA–dentin interface exhibited the highest mean gap width (9.88 ± 4.12 μm), which was significantly greater than that of the NeoPUTTY–dentin interface (7.44 ± 1.46 μm; *p* = 0.006), and greater, though not significantly, than the Biodentine–dentin interface (8.84 ± 3.99 μm; *p* = 0.356) (Fig. 9). The difference between Biodentine and NeoPUTTY was also statistically non-significant (*p* = 0.124).

**Table 3:** Values of the interphase between tested material and dentin gap area measurements in µM

	Mean	Std. Deviation	Std. Error	95% Confidence Interval for Mean		Minimum	Maximum
				Lower Bound	Upper Bound		
Biodentine	8.8430	3.98878	0.56983	7.6973	9.9888	3.02	18.61
MTA	9.8857	4.12668	0.70772	8.4459	11.3256	3.01	19.16
NeoPUTTY	7.4453	1.46393	0.22325	6.9947	7.8958	4.71	11.91
Total	8.6474	3.50067	0.31186	8.0302	9.2646	3.01	19.16

## Discussion

The principal aim of the pulpotomy technique is to maintain a functional primary tooth without symptoms until its natural exfoliation occurs [6]. The procedural success is largely dependent on the effectiveness of the coronal restoration in preventing bacterial penetration [29]. The colonization of bacteria in the root canal can elicit an inflammatory response when bacterial byproducts, including exotoxins or endotoxins, infiltrate the periradicular tissues [19, 30]. Bacterial colonization within dentinal tubules and subsequent biofilm formation remain critical challenges in pulpotomy [31], this emphasizes the necessity of using biocompatible materials that provide both an effective seal and antimicrobial properties [15, 32].

The growing importance of calcium silicate in restorative dentistry can be attributed to its excellent biocompatibility, which supports the formation of a high-quality dentin bridge, as well as its ability to create an impermeable barrier over the pulp [28, 33]. In this study, the performance of NeoPUTTY was compared with Biodentine and MTA in pulpotomized mandibular second primary molars, with regard to their sealing ability and antibacterial effects.

In this study, the bacterial leakage model was adopted due to its higher biological and clinical relevance compared to conventional methods such as dye penetration, turbidity, or radioactive tracer tests, which are often influenced by factors like dye molecular size, immersion time, or air entrapment and may not accurately replicate microbial pathways [18, 34–36]. Nevertheless, bacterial leakage models also have limitations, particularly in tracing the exact routes of bacterial invasion. To overcome these drawbacks and enhance the reliability of our findings, CLSM and ESEM were incorporated as complementary techniques. CLSM enabled a direct, non-destructive three-dimensional evaluation of bacterial viability within dentinal tubules, while ESEM allowed detailed visualization of interfacial gaps under hydrated conditions without extensive specimen preparation.

By adopting a bacterial leakage model and integrating confocal laser scanning microscopy (CLSM) and environmental scanning electron microscopy (ESEM), our study offered a more comprehensive and reliable assessment than conventional methods such as dye penetration or turbidity tests, which have well-documented limitations [18, 34–36]. For instance, dye penetration tests are influenced by dye molecular size, pH, immersion time, and the presence of air entrapment, which compromise their reliability [36]. Considering the need for experimental models that accurately replicate microbial colonization pathways, *Enterococcus faecalis* was selected as the test organism. *E. faecalis* is the most frequently isolated species in persistent endodontic infections and is well known for its strong capacity for biofilm formation and tissue invasion, making it a highly relevant and an ideal model organism for studying bacterial invasion in the present study [16, 17, 25, 37, 38]. CLSM and ESEM were incorporated as complementary techniques. However, a major limitation of CLSM is that it cannot directly visualize non-fluorescent materials. Therefore, the use of fluorescent dyes, is necessary to visualize the penetration of materials [15].

In the current study, all the positive control samples exhibited fluorescence, confirming the necessity of pulp dressing material, while no fluorescence was observed in the negative control samples, confirmed the reliability of the methodology adopted. The nail polish totally prevented microleakage, with bacterial contamination only penetrating the coronal portion of the teeth. This validates the methodology adopted in the current study [15, 17, 25].Additionally, all experimental procedures were carried out by a single calibrated operator following a standardized protocol to ensure methodological consistency and minimize bias and variability.

According to CLSM analysis, bacterial viability was significantly affected by the type of material used. We found that MTA exhibited the highest bacterial leakage compared to Biodentine and NeoPUTTY (*p* < 0.0001, for both), with no significant difference between the latter two (*p* = 0.97), although NeoPUTTY demonstrated the lowest live bacterial load. ESEM further confirmed that NeoPUTTY presented fewer interfacial gaps than both Biodentine and MTA, indicating its superior sealing ability. Therefore, the null hypothesis of this study was rejected.

Our findings are consistent with previous studies [39–45] demonstrated that Biodentine exhibits superior sealing ability and antibacterial properties compared with MTA. This could be attributed to several factors; first, its smaller particle size and lower porosity enable closer adaptation to dentinal walls thus enhancing its marginal sealing, as confirmed by SEM [39, 40] and computed microtomography [41] observations, which demonstrated that Biodentine adapts more closely to dentinal walls than MTA. In another study, it was noted that Biodentine’s sealing ability closely resembled that of apatite crystals when observed under a SEM [42].

Additionally, when Biodentine comes in contact with dentine it leads to the formation of tag-like structures alongside an interfacial layer called the “mineral infiltration zone,” where the alkaline caustic effect of calcium silicate cements hydration products degrades the collagenous component of interfacial dentine. This promotes mineral infiltration into dentin and the subsequent formation of a stable hydroxyapatite layer at the interface, creating a biological seal [43]. Han and Okiji [44] showed that calcium and silicon ion uptake into dentin leading the formation of tag-like structures in Biodentine was higher than MTA. Furthermore, the fast setting time of Biodentine (approximately 12 min) allows for earlier sealing of the dentin–material interface, thereby minimizing the risk of microleakage and bacterial contamination [45].

The antibacterial action of Biodentine is mainly due to its alkalinity and calcium hydroxide release during cement hydration process, which generates a colloidal gel that raises pH to around 12.5, inhibiting bacterial growth and disinfecting the surrounding dentin [46]. It has also been reported to inhibit microbial adhesion and biofilm formation in vivo [47]. This also comes in agreement with previous studies [17, 39, 48] where Biodentine demonstrated superior effectiveness against *Enterococcus faecalis* compared to MTA.

Although there was no statistically significant difference between Neoputty and Biodentine regarding microleakage (*p* = 0.124) and bacterial viability (*p* = 0.972), the least microleakage and lowest bacterial viability were noted in the Neoputty group could be attributed to its premixed formulation, ultrafine tricalcium and dicalcium silicates, and bioactive organic carrier contribute to hydroxyapatite formation, improved sealing and antibacterial activity. Furthermore, its firm, non-tacky consistency, resistance to washout than Biodentine, and notable bioactivity. This is consistent with the findings of [49, 50], who demonstrated its enhanced penetration into dentinal tubules than Biodentine and NeoMTA. Similarly, Acharya et al. [51], in a study comparing Modified NeoPutty MTA^®^, Biodentine, and Calcium Hydroxide in indirect pulp therapy of deciduous teeth, highlighted the favorable properties and superior clinical performance of NeoPutty MTA compared to the other materials evaluated.

In contrast, Acharya et al. reported limited antibacterial activity of NeoPUTTY in zone of inhibition assays against *E. faecalis*, which improved when antibiotics [52] were added. While, Eid et al. [53] found that it exhibited greater microleakage than ProRoot MTA and Biodentine in dye penetration tests. These differences appear to be method-dependent, since dye penetration models can overestimate leakage due to the very small molecular size of dye particles.

Previous literature has reported conflicting data regarding MTA’s antibacterial effectiveness. Some studies have shown limited activity [54, 55], while others demonstrated antibacterial effects against *E. faecalis* [15, 56]. Freshly mixed MTA was shown to possess stronger antibacterial properties, and Biodentine has been found to exhibit comparable activity [57].

On the other hand, MTA has been criticized for its drawbacks, including long setting time, handling difficulties, surface disintegration that may compromise marginal adaptation and relatively high cytotoxicity in its freshly mixed stage [54, 55]. Reports on MTA’s antibacterial activity remain inconsistent, with some studies showing limited effects against *E. faecalis* [56, 57], while others demonstrate measurable antibacterial activity, particularly in freshly mixed formulations [58, 59].

Anatomical variability appears to significantly influence bacterial infiltration. Higher bacterial loads were consistently observed in the mesial and apical areas, likely due to the complex morphology of the mesial pulp horns and the pulp chamber floor. These regions often contain accessory foramina and irregular surfaces, which hinder effective material adaptation and promote bacterial ingress [60, 61]. These findings highlight the critical importance of selecting materials with superior adaptability and optimizing placement techniques to ensure effective coronal sealing.

The presence of microgaps at the material–dentine interface was found to be clinically significant, as these voids facilitate fluid and bacterial penetration, potentially leading to postoperative sensitivity and secondary caries [16, 28]. ESEM results confirmed CLSM findings, showing that NeoPUTTY had significantly the least microgap formation (7.44 ± 1.46 μm) (*p* = 0.006) compared to MTA, which displayed the highest gap values (9.88 ± 4.12 μm), followed by Biodentine (8.84 ± 3.99 μm). This further supports the superior sealing ability and marginal adaptation of NeoPUTTY, which may reduce the risk of microleakage-related complications.

### Limitations

This study has several limitations that should be acknowledged. First, it was conducted under in vitro conditions that cannot fully replicate the complex biological environment of the oral cavity. Second, only a single bacterial species (*E. faecalis*) was tested, whereas clinical infections typically involve multispecies biofilms. Third, the relatively small sample size and short-term evaluation; thus, long-term changes in antibacterial activity, sealing, and marginal adaptation were not assessed. Finally, outcomes may have been influenced by variables such as dentin characteristics, smear layer presence, laboratory conditions and sample preparation. Also, variations in crown height could have influenced the final thickness of the applied materials, even though standardized access cavity dimensions were used. Further clinical studies with larger sample sizes, long-term follow-up, and inclusion of first permanent molars —commonly treated with pulpotomy— are required to confirm these findings and optimize material selection in pediatric dentistry.

## Conclusion

Within the limitations of this in vitro study, Biodentine and NeoPUTTY have significant antibacterial activity, sealing, and marginal adaptation compared to MTA. CLSM and ESEM imaging confirmed that interfacial gaps were directly associated with higher bacterial penetration and surface irregularities, particularly in the MTA group. The enhanced performance of Biodentine and NeoPUTTY may be related to their bioactive composition and improved adaptability. These results provide valuable guidance for clinical decision-making in pediatric dentistry, emphasizing the importance of material selection and interfacial integrity in achieving long-term success.

## Data Availability

The datasets used and/or analyzed during the current study are available from the corresponding author on reasonable request.

## References

[1] Duggal MS, Nooh A, High A. Response of the primary pulp to inflammation: a review of the Leeds studies and challenges for the future. Eur J Paediatr Dent. 2002;3:111–4. 12870998

[2] Rodd HD, Waterhouse PJ, Fuks AB, Fayle SA, Moffat MA. Pulp therapy for primary molars. Int J Paediatr Dent. 2006;16:15–23. 16939452 10.1111/j.1365-263X.2006.00774.x

[3] Waterhouse PJ, Whitworth JM, Camp JH, Fuks AB.Pediatric endodontics: endodontic treatment for the primary and young permanent dentition.In: Cohen S, Hargreaves KM, editors.Cohen’s Pathways of the Pulp.10th ed. St. Louis: Mosby Elsevier; 2011. p. 808–857.

[4] American Academy of Pediatric Dentistry. Pulp therapy for primary and immature permanent teeth. In: The Reference Manual of Pediatric Dentistry. Chicago: American Academy of Pediatric Dentistry; 2023. p. 457–465.

[5] Kim CH, Bae JS, Kim I-H, Song JS, Choi H-J, Kang C-M. Prognostic factors for the survival of primary molars following pulpotomy with mineral trioxide aggregate: a retrospective cohort study. Clin Oral Investig. 2021;25:1797–804. 32754786 10.1007/s00784-020-03482-3

[6] Holan G, Fuks AB, Ketlz N. Success rate of formocresol pulpotomy in primary molars restored with stainless steel crown vs amalgam. Pediatr Dent. 2002;24:212–6. 12064493

[7] Hench LL. The story of Bioglass^®^. J Mater Sci Mater Med. 2006;17:967–78. 17122907 10.1007/s10856-006-0432-z

[8] Torabinejad M, Chivian N. Clinical applications of mineral trioxide aggregate. J Endod. 1999;25:197–205. 10321187 10.1016/S0099-2399(99)80142-3

[9] Musale PK, Soni AS. Clinical pulpotomy trial of Copaifera langsdorffii oil resin versus formocresol and white mineral trioxide aggregate in primary teeth. Pediatr Dent. 2016;38:E5–12. 27097854

[10] Vilella-Pastor S, Sáez S, Veloso A, Guinot-Jimeno F, Mercadé M. Long-term evaluation of primary teeth molar pulpotomies with Biodentine and MTA: a CONSORT randomized clinical trial. Eur Archives Pediatr Dentistry. 2021;22:685–92. 10.1007/s40368-021-00616-3 33683572

[11] Chak RK, Singh RK, Mutyala J, Killi NK. Clinical radiographic evaluation of 3Mixtatin and MTA in primary teeth pulpotomies: A randomized controlled. Int J Clin Pediatr Dent. 2022;15(Suppl 1):S80. 35645497 10.5005/jp-journals-10005-2216 PMC9108818

[12] Guo J, Zhang N, Cheng Y. Comparative efficacy of medicaments or techniques for pulpotomy of primary molars: a network meta-analysis. Clin Oral Investig. 2023;27:91–104. 36580161 10.1007/s00784-022-04830-1 PMC9876877

[13] Elsayed N, Hegazy EM, Amin MH, Farag MS, Omer SMM. Efficacy of combination of Biodentine and Simvastatin as pulp capping materials in vital pulpotomy of primary molars: randomized clinical trial. Dent Sci Updates. 2023;4:205–18.

[14] Selvendran KE, Ahamed AS, Krishnamurthy M, Kumar VN, Raju VG. Comparison of three different materials used for indirect pulp capping in permanent molars: an: in vivo: study. J Conservative Dentistry. 2022;25:68–71. 10.4103/jcd.jcd_551_21 PMC9200191 35722078

[15] Elbahary S, Haj-Yahya S, Khawalid M, Tsesis I, Rosen E, Habashi W, et al. Effects of different irrigation protocols on dentin surfaces as revealed through quantitative 3D surface texture analysis. Sci Rep. 2020;10:22073. 33328515 10.1038/s41598-020-79003-9 PMC7744534

[16] Snigdha NTS, Kamarudin A, Baharin F, Ghani NRNA, bin Yhaya MF, Ahmad WMAW, et al. Evaluation of bacterial leakage and marginal adaptation of the bioceramics pulp dressing materials: an invitro study. BMC Oral Health. 2023;23:462. 37420224 10.1186/s12903-023-03129-1 PMC10329390

[17] Tsesis I, Elbahary S, Venezia NB, Rosen E. Bacterial colonization in the apical part of extracted human teeth following root-end resection and filling: a confocal laser scanning microscopy study. Clin Oral Investig. 2018;22:267–74. 28349219 10.1007/s00784-017-2107-1

[18] Elbahary S, Gitit Z, Flaisher-Salem N, Azem H, Shemsesh H, Rosen E, et al. Influence of irrigation protocol on peroxide penetration into dentinal tubules following internal bleaching: A confocal laser scanning microscopy study. J Clin Pediatr Dentistry. 2021;45:253–8. 10.17796/1053-4625-45.4.6 34534304

[19] Guelmann M, Bookmyer KL, Villalta P, García-Godoy F. Microleakage of restorative techniques for pulpotomized primary molars. J Dent Child. 2004;71:209–11. 15871455

[20] El Makawi Y, Khattab N. In vitro comparative analysis of fracture resistance of lithium disilicate endocrown and prefabricated zirconium crown in pulpotomized primary molars. Open Access Maced J Med Sci. 2019;7:4094. 32165959 10.3889/oamjms.2019.864 PMC7061377

[21] Faul F, Erdfelder E, Lang A-G, Buchner A. G* power 3: A flexible statistical power analysis program for the social, behavioral, and biomedical sciences. Behav Res Methods. 2007;39:175–91. 17695343 10.3758/bf03193146

[22] Sandhu SV et al. Sterilization of extracted human teeth: a comparative analysis. J Oral Biology Craniofac Res. 2012;2(3):170–5. 10.1016/j.jobcr.2012.09.002 PMC3942122 25737861

[23] Moskovitz M, Tickotsky N. Pulpectomy and root canal treatment (RCT) in primary teeth: techniques and materials. In: Fuks AB, Peretz B, editors. Pediatric endodontics: current concepts in pulp therapy for primary and young permanent teeth. Cham: Springer; 2016. p. 71–101.

[24] Formosa LM, Mallia B, Camilleri J. The microstructure and surface morphology of radiopaque tricalcium silicate cement exposed to different curing conditions. Dent Mater. 2012;28:584–95. 10.1016/j.dental.2012.02.006 22410112

[25] Elbahary S, Aharonian S, Azem H, Peretz B, Mostinski O, Blumer S. Bacterial colonization and proliferation in primary molars following the use of the hall technique: A confocal laser scanning microscopy study. Children. 2023;10:457. 36980014 10.3390/children10030457 PMC10047319

[26] Schindelin J, Arganda-Carreras I, Frise E, Kaynig V, Longair M, Pietzsch T, et al. Fiji: an open-source platform for biological-image analysis. Nat Methods. 2012;9:676–82. 22743772 10.1038/nmeth.2019 PMC3855844

[27] Wells JD, Pashley DH, Loushine RJ, Weller RN, Kimbrough WF, Pereira PN. Intracoronal sealing ability of two dental cements. J Endod. 2002;28:443–7. 12067125 10.1097/00004770-200206000-00006

[28] Yavuz Y, Kotanli S, Doğan MS, Doğan K. Comparisons of microleakage and scanning electron microscope (SEM) analyses of the use of different pulp coverage materials. Makara J Health Res. 2022;26(2):140–45.

[29] Ray HA, Trope M. Periapical status of endodontically treated teeth in relation to the technical quality of the root filling and the coronal restoration. Int Endod J. 1995;28:12–8. 7642323 10.1111/j.1365-2591.1995.tb00150.x

[30] Yamasaki M, Nakane A, Kumazawa M, Hashioka K, Horiba N, Nakamura H. Endotoxin and gram-negative bacteria in the rat periapical lesions. J Endod. 1992;18:501–4. 1289475 10.1016/S0099-2399(06)81351-8

[31] Chivatxaranukul P, Dashper SG, Messer HH. Dentinal tubule invasion and adherence by Enterococcus faecalis. Int Endod J. 2008;41:873–82. 18822013 10.1111/j.1365-2591.2008.01445.x

[32] Gartner AH, Dorn SO. Advances in endodontic surgery. Dent Clin North Am. 1992;36:357–78. 1572504

[33] Reis M, de Scarparo S, Signor RK, Bolzan B, Steier JT, de Figueiredo L. Pulp capping with mineral trioxide aggregate or biodentine: a comparison of mineralized barrier formation and inflammatory and degenerative events. Braz Oral Res. 2021;35:e118. 34878073 10.1590/1807-3107bor-2021.vol35.0118

[34] del Carpio-Perochena A, Bramante CM, de Andrade FB, Maliza AGA, Cavenago BC, Marciano MA, et al. Antibacterial and dissolution ability of sodium hypochlorite in different pHs on multi-species biofilms. Clin Oral Investig. 2015;19:2067–73. 25715919 10.1007/s00784-015-1431-6

[35] Louwakul P, Saelo A, Khemaleelakul S. Efficacy of calcium oxide and calcium hydroxide nanoparticles on the elimination of Enterococcus faecalis in human root dentin. Clin Oral Investig. 2017;21:865–71. 27129586 10.1007/s00784-016-1836-x

[36] WU M-K, WESSELINK PR. Endodontic leakage studies reconsidered. Part I. Methodology, application and relevance. Int Endod J. 1993;26:37–43. 8473032 10.1111/j.1365-2591.1993.tb00540.x

[37] Brosco VH, Bernardineli N, Torres SA, Consolaro A, Bramante CM, de Moraes IG, et al. Bacterial leakage in obturated root canals—part 2: a comparative histologic and microbiologic analyses. Volume 109. Oral Radiology, and Endodontology: Oral Surgery, Oral Medicine, Oral Pathology; 2010. pp. 788–94. 10.1016/j.tripleo.2009.11.036 20416539

[38] Schleifer KH, Kilpper-Bälz R. Transfer of Streptococcus faecalis And Streptococcus faecium to the genus Enterococcus nom. rev. As Enterococcus faecalis comb. Nov. And Enterococcus faecium comb. Nov. Int J Syst Evol Microbiol. 1984;34:31–4.

[39] Samuel A, Asokan S, Geetha Priya PR, Thomas S. Evaluation of sealing ability of Biodentine™ and mineral trioxide aggregate in primary molars using scanning electron microscope: A randomized controlled in vitro trial. Contemp Clin Dent. 2016;7(3):322–5. 27630495 10.4103/0976-237X.188547 PMC5004544

[40] Kumbhar AJ, Kamat SB, Hugar SI, Nanjannawar GS, Kulkarni NR. Comparative evaluation of marginal adaptation of mineral trioxide aggregate, Biodentine, and geristore as a root end filling material: an in vitro scanning electron microscope study. J Conserv Dent Endod. 2023;26(4):447–52. 37705552 10.4103/jcd.jcd_55_21 PMC10497075

[41] Guerrero F, Berástegui E. Porosity analysis of MTA and Biodentine cements for use in endodontics by using micro-computed tomography. J Clin Exp Dent. 2018;10(3):e237–40. 29721224 10.4317/jced.54688 PMC5923892

[42] Jantarat J, Ritsayam S, Banomyong D, et al. Early and 24-hour shear bond strength to dentine of three calcium silicate based pulp capping materials. Mah Dent J. 2018;38:177–83.

[43] Paul NM. Evaluation of microleakage of Biodentine as a dentin substitute compared to Fuji II LC in cervical lining restorations: an in vitro study [master’s thesis]. Bengaluru: Rajiv Gandhi University of Health Sciences; 2016.

[44] Han L, Okiji T. Uptake of calcium and silicon released from calcium silicate-based endodontic materials into root Canal dentine. IntEndod J. 2011;44:1081–7. 10.1111/j.1365-2591.2011.01924.x 21777256

[45] Behr M, Rosentritt M, Loher H, Kolbeck C, Trempler C, Stemplinger B. Changes of cement properties caused by mixing errors: the therapeutic range of different cement types. Dent Mater. 2008;24:1187–93. 18372038 10.1016/j.dental.2008.01.013

[46] Estrela C, Cintra LTA, Duarte MAH, Rossi-Fedele G, Gavini G, Sousa-Neto MD. Mechanism of action of bioactive endodontic materials. Braz Dent J. 2023;34(1):1–11. 36888836 10.1590/0103-6440202305278 PMC10027099

[47] Al-Ahmad A, Haendel M, Altenburger MJ, Karygianni L, Hellwig E, Wrbas KT, et al. Biodentine inhibits the initial microbial adhesion of oral microbiota in vivo. Antibiotics. 2022;12(1):4. 36671205 10.3390/antibiotics12010004 PMC9855060

[48] Esteki P, Jahromi MZ, Tahmourespour A. In vitro antimicrobial activity of mineral trioxide aggregate, biodentine, and calcium-enriched mixture cement against Enterococcus faecalis, Streptococcus mutans, and Candida albicans using the agar diffusion technique. Dent Res J. 2021;18(3):184–90. PMC8122692 34084290

[49] De S, Naik NS, Sharma S, Vashisth P, Dua R, Maheshwari P. Stereomicroscopic Evaluation of Sealing Ability of Three Different Furcal Perforation Repair Materials: An In vitro Study. Contemp Clin Dent. 2024;15(4):259–64. 10.4103/ccd.ccd_253_24 PMC11749040 39845621

[50] Ozdemir M, Oncu A. Penetration of Biodentine, NeoMTA 2, and NeoPUTTY into dentinal tubules in primary tooth pulpotomy: a confocal laser scanning microscopy analysis. BMC Oral Health. 2025;25:727. 40375201 10.1186/s12903-025-06065-4 PMC12083007

[51] Acharya S, Gurunathan D, Assiry AA, et al. Comparison of modified NeoPutty MTA^®^, Biodentine, and calcium hydroxide in indirect pulp therapy in deciduous teeth: an in vivo clinical study. Int J Clin Pediatr Dent. 2024;17(9):1025–9. 39664819 10.5005/jp-journals-10005-2953 PMC11628681

[52] Acharya S, Gurunathan D, Sahoo D, Singh B, Sahoo A, Acharya S. Comparative evaluation of the antimicrobial activity of NeoPutty MTA and modified NeoPutty MTA: an in vitro study. J Int Soc Prev Community Dent. 2023;13(6):493–9. 38304536 10.4103/jispcd.JISPCD_68_23 PMC10829285

[53] Eid BM, Alarfaj BA, Abdelaal HM, et al. Microleakage assessment of calcium Silicate-based Root-end filling materials using dye penetration: an in vitro study. World J Dent. 2023;14(5):435–9.

[54] Khandelwal A, Karthik J, Nadig RR, Jain A. Sealing ability of mineral trioxide aggregate and Biodentine as the root-end filling material using two different retro-preparation techniques: an in vitro study. Int J Contemp Dent Med Rev. 2015;1–6.

[55] Hondares TC. An evaluation of the in vitro antibacterial, biocompatibility, and mineralization properties of six calcium silicate-based pulp capping materials MS thesis. The University of Alabama at Birmingham, 2022.

[56] Estrela C, Bammann LL, Estrela CR, Silva RS, Pécora JD. Antimicrobial and chemical study of MTA, Portland cement, calcium hydroxide paste, sealapex and dycal. Braz Dent J. 2000;11:3–9. 11210272

[57] Parirokh M, Asgary S, Eghbal MJ, Kakoei S, Samiee M. A comparative study of using a combination of calcium chloride and mineral trioxide aggregate as the Pulp-capping agent on dogs’ teeth. J Endod. 2011;37:786–8. 21787489 10.1016/j.joen.2011.03.010

[58] Tanomaru-Filho M, Tanomaru JMG, Barros DB, Watanabe E, Ito IY. In vitro antimicrobial activity of endodontic sealers, MTA-based cements and Portland cement. J Oral Sci. 2007;49:41–5. 17429181 10.2334/josnusd.49.41

[59] Koruyucu M, Topcuoglu N, Tuna EB, Ozel S, Gencay K, Kulekci G, et al. An assessment of antibacterial activity of three pulp capping materials on Enterococcus faecalis by a direct contact test: an in vitro study. Eur J Dent. 2015;09:240–5. 10.4103/1305-7456.156837 PMC4439853 26038657

[60] KRASNER P, RANKOW H. Anatomy of the Pulp-Chamber floor. J Endod. 2004;30:5–16. 14760900 10.1097/00004770-200401000-00002

[61] Gangwar A, De S, Dua R, Maheshwari P, Ghosh J, Kumari S. Assessment of pulp chamber morphology of primary maxillary and mandibular molars using spiral computed tomography: an analytical study. Int J Clin Pediatr Dent. 2025;18:431–5. 40469830 10.5005/jp-journals-10005-3062 PMC12131055

